# Supplementary material for: Nutritional and Phytochemical Characterization of Radish Leaves: A Comprehensive Overview
Source: Foods. 2025 Sep 20;14(18):3270. doi: 10.3390/foods14183270 (PMC12469553; doi:10.3390/foods14183270)

Supplementary Material Figure S1. Diagrams of the roots and leaves of three common types of radishes.

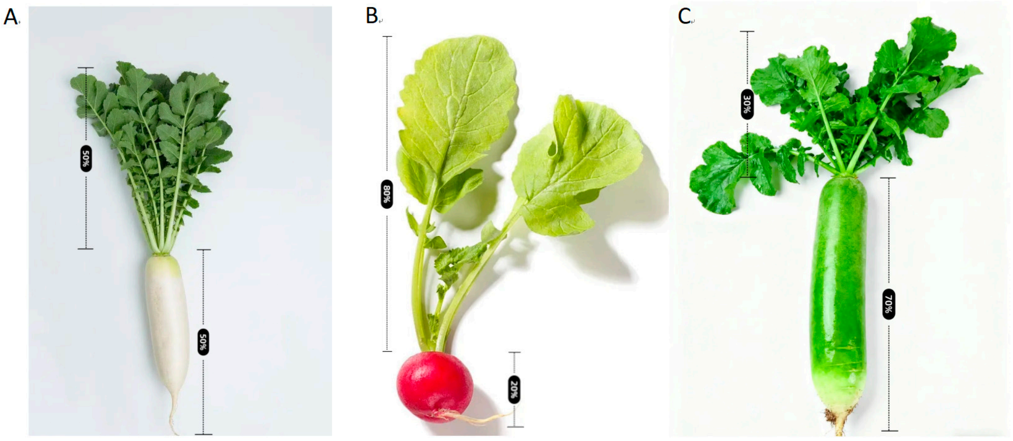

Supplement: Supplementary file 1 [file foods-14-03270-s001.zip › foods-3849469-supplementary.pdf]
